# Effectiveness of mindfulness-based interventions on burnout and self-compassion among critical care nurses caring for patients with COVID-19: a quasi-experimental study

**DOI:** 10.1186/s12912-023-01466-8

**Published:** 2023-09-06

**Authors:** Sahar Younes Othman, Nagia I. Hassan, Alaa Mostafa Mohamed

**Affiliations:** 1 Critical Care and Emergency Nursing Department, Faculty of Nursing, Damanhour University, El-Beheira, Egypt; 2 Psychiatric and Mental Health Nursing Department, Faculty of Nursing, Damanhour University, El-Beheira, Egypt

**Keywords:** Mindfulness-based intervention, Burnout, Compassion, Critical care nurses, Coronavirus disease 2019 (COVID-19)

## Abstract

**Background:**

Workloads in intensive care units (ICUs) have increased and extremely challenging ethical dilemmas were generated by the coronavirus disease 2019 (COVID-19) pandemic. ICU nurses experience high-stress levels and burnout worldwide. Egyptian studies on the effectiveness of mindfulness-based intervention (MBI) among ICU nurses are limited, although MBI has been shown to reduce stress and burnout.

**Methods:**

This quasi-experimental study included 60 nurses working in three hospitals in El-Beheira, Egypt. Participants were randomly allocated to one of the two groups: intervention or control (30 participants per group). The participants in the intervention group (MBI) received 8 MBI sessions, whereas the control group received no intervention. The Maslach Burnout Inventory, the Five-Facet Mindfulness Questionnaire (FFMQ), and the Self-Compassion Scale were used to assess the outcomes. Additionally, demographic and workplace data were collected.

**Results:**

The post-test score of emotional exhaustion after MBI for 8 weeks significantly decreased in the MBI group to 15.47 ± 4.44 compared with the control group with 32.43 ± 8.87 (*p* < 0.001).

The total Self-Compassion Scale significantly increased because of the mindfulness sessions 94.50 ± 3.83 for the MBI group vs. 79.00 ± 4.57 for the control group (*p* < 0.001). The post-test score of the FFMQ significantly increased to 137.03 ± 5.93, while the control group’s score decreased to 114.40 ± 7.44, following the MBI sessions (*p* < 0.001). As determined by Cohen’s d test, the effect size of MBI training is quite large, on the three burnout scale dimensions (emotional exhaustion, depersonalization, and personal achievement), as well as the total score of the mindfulness and self-compassion scales.

**Conclusion:**

This study provides preliminary evidence that MBI sessions were effective in reducing emotional exhaustion and depersonalization and increasing levels of mindfulness and self-compassion among critical care nurses.

## Background

Working in an intensive care unit (ICU) introduces a real-life stressor that is difficult to change due to the nature of the work [1, 2]. The critical care environment is difficult and uncertain under normal conditions [3, 4]. The nature of critical illness and the relational aspects of working in large multidisciplinary healthcare teams add to the complexity. The uncertainty about the prognosis and outcomes of critical care interventions is common. Moreover, complex moral and ethical debates in the ICU are common because end-of-life care in the ICU frequently coincides with deliberate and informed decisions to withdraw life-sustaining measures [4, 5]. Therefore, critical care nurses (CCNs) frequently face uncertainty, heavy workloads, and complexity in their work, making them prone to burnout and emotional distress [6]. Despite some of these issues and challenges are globally widespread among ICU services, others are unique to the ICU situation in Egypt. In Egypt, unnecessary long stays of patients who have survived the acute phase or unnecessary admission of patients who merely need intermediate care or monitoring increases ICU bed occupancy and exhaust health care workers (HCWs) [7].

The coronavirus disease 2019 (COVID-19) pandemic offers a unique opportunity to magnify these common experiences in real time [8]. The recent COVID-19 pandemic heightened nurses’ anxiety and stress by raising their concerns about the disease and its potential to infect them or their loved ones [9]. Staffing shortages, a lack of personal protective equipment, and an unexpected increase in the number of patients with COVID-19 in critical care units (CCUs) increase the risk of infection for CCNs [10]. Anxiety, depression, post-traumatic stress disorder (PTSD), moral distress, and burnout are all risk factors for high acuity and mortality of critically ill patients with COVID-19 when resources are inadequate [11–13]. Nurses may experience moral distress or burnout if they feel helpless, have little control, lack resources or support, have a demanding job, or are subjected to excessive demands [14].

Numerous global studies have revealed the significant negative impact of the COVID-19 pandemic on the mental health of CCNs. Heesakkers et al. reported the frequency of anxiety, depression, and PTSD among CCNs as 27.0%, 18.6%, and 22.2%, respectively [15]. In Egypt, the rate of HCWs experiencing mental symptoms during the COVID-19 pandemic was greater than that before the outbreak. A study by Baraka et al. in Egypt [16] revealed that 62% of CCNs experienced severe anxiety, 38.5% suffered from severe stress, and 34.5% exhibited moderate depression while caring for patients with COVID-19. Additionally, Arafa et al. [17] revealed that front-line healthcare workers (HCWs) in Egypt and Saudi Arabia experienced depression (69%) and anxiety (58.9%), as well as stress (55.9%) and inadequate sleep (37.3%) during the COVID-19 pandemic.

The high estimated incidence of mental health disorders among Egyptian HCWs may be associated with several factors. Egyptian HCWs faced challenging workloads, especially during the COVID-19 pandemic. Hospitals in Egypt, can be either public or private, and a large number of HCWs operate in both settings [18]. According to the most recent census in 2017, there were 22.3 nurses for every 10,000 people [19]. The shortage of nurses, particularly qualified CCNs, combined with a lack of medical supplies reflects poor ICU situations in Egypt, which is highly correlated with the deterioration of CCNs’ mental health during the pandemic [20].

Hence, the need for efficient strategies to alleviate stress in the HCW sector is growing. Burnout and stress in CCNs can be reduced through a variety of methods. Mindfulness-based intervention (MBI) is one strategy proposed to aid CCNs [21]. Mindfulness is the intentional and nonjudgmental awareness of present-moment thoughts, feelings, and bodily sensations [22]. Mindfulness is a state of consciousness that helps the mind and body find balance and harmony, which are key components of a state of well-being [23]. The most researched MBIs, such as mindfulness-based stress reduction (MBSR) [22] and mindfulness-based cognitive therapy [24], are 8-week programs with weekly 2-h and 30-min group meetings and an intensive day in between.

The concept of mindfulness can be viewed as a set of abilities that include both state and trait domains. Trait mindfulness refers to a stable attitude or individual difference in the tendency to be mindful in daily life, irrespective of the context or circumstance. State mindfulness is a temporary state of consciousness where an individual is aware of their present moment experience, including thoughts, feelings, and body sensations, without judgment [25]. The trait domain of mindfulness is more stable over time but may be reinforced with repeated mindfulness training, whereas state mindfulness is frequently increased in the present and shortly following mindfulness training [26]. Both elements are assessed using self-report scales, such as the State Mindfulness Scale for state mindfulness and the Five Facets Mindfulness Questionnaire (FFMQ) for trait mindfulness [27]. In this study, we adopted trait mindfulness to investigate its effect on burnout, mindfulness, and self-compassion among CCNs.

Studies on the spread of clinical models that are based on mindfulness have led us to believe that it plays a key role in emotional self-regulation [28]. Therefore, by using self-processing mechanisms and adjusting awareness of one’s identity, emotional states, behavioral patterns, and relationships with others, mindfulness may help reduce suffering and build a healthy mind [29]. Compassion is the emotional and empathic awareness of suffering of another person that makes it possible to deal with suffering of other people, like that of patients and carers [30]. Being compassionate means being aware of own and other people’s suffering and having the urge or motivation to end it [31]. Compassion has been associated with coping with distressing experiences during extraordinary events, such as pandemics, particularly among HCWs [21, 32, 33].

MBI reduces stress and improves well-being in various populations and contexts. Guillaumie et al. [34] revealed that mindfulness improves nurses’ mental health. Ghawadra et al. reported that MBSR reduced nurses’ psychological distress [35]. Additionally, Matos et al. [32] revealed that MBI reduced emotional burden and burnout in nurses with burnout syndrome. The intervention is practical, economical, and time-saving, especially for busy CCNs. However, CCN studies are scarce [36]. Only a few studies were conducted on CCN mindfulness during the COVID-19 pandemic. Based on the findings from mindfulness protocols, we hypothesize the usefulness of MBIs in reducing burnout and psychological distress among CCNs working in pandemic conditions. Much remains to be learned about the best MBI, such as measuring their psychological well-being and overall effectiveness in improving the psychological health of CCNs, despite the growing body of research on the benefits of MBI for nurses.

### Study aim

This study aims to examine the effectiveness of MBIs (MBSR) on burnout, mindfulness, and self-compassion among CCNs caring for patients with COVID-19. Our research addresses the following question: what is the effect of MBIs on burnout, mindfulness, and self-compassion among CCNs who are caring for patients with COVID-19? We hypothesized that CCNs who participate in MBIs report lower levels of burnout, higher levels of mindfulness, and greater self-compassion compared with CCNs who do not participate in MBIs.

## Methods

### Study design and setting

This quasi-experimental prospective study conducted pre- and post-test assessments in the intervention and control groups. This study used three ICUs of three Ministry of Health-affiliated COVID-19 quarantine hospitals in El-Beheira Governorate of Egypt. The selection of ICUs and hospitals was based on a purposive sampling approach according to certain criteria, such as geographic location and availability of nurses who were willing to participate. As we were conducting the study during the COVID-19 pandemic, several nurses were already working long hours and did not have the time or desire to participate in research-related activities.

### Participants

Nurses who directly cared for patients with COVID-19 were recruited using convenient sampling. The study included nurses who volunteered to participate. Inclusion criteria were being a full-time registered nurse working in the COVID-19 ICU, having at least 6 months of ICU experience, owning a smartphone, and agreeing to participate. Exclusion criteria are previous participation in MBI training programs, receiving psychosocial or psychiatric treatment, or not completing the questionnaire. Participants were randomly assigned using a random number table to either the intervention (MBI) or control group (*n* = 30 in each). To avoid cross-contamination, the intervention was given to the experimental group at a different time and place than control group. Participants were also urged to refrain from sharing intervention materials with the control group. We emphasized the need for confidentiality and keeping the subject within the research group.

### Sample size

Sample size calculation was performed using the comparison of the FFMQ between the intervention and control groups. As reported in a previous study [37], the mean ± standard deviation (SD) of the FFMQ in the intervention group was 140.1 ± 14.2, whereas that in control group was 114.8 ± 20.4. Using the highest SD of the control, we calculated that the minimum proper sample size was 30 participants in each group for an able detection of a real difference of 15 points with 80% power at α = 0.05 level using a Student’s t-test for independent samples. Sample size calculation was performed using G*Power software version 3.1.9.6 for MS Windows (Franz Faul, Kiel University, Germany). The following equation was used:

$$\mathrm{n}=2{\left({\mathrm{Z}}_{1-\mathrm{a}/2}+{\mathrm{Z}}_{1-\upbeta }\right)}^{2/{\mathrm{d}}^{2}}$$

where “n” is the calculated sample size; Z1-α/2 is the Z score for the significance level 0.05; Z1-β is the Z score of 80% power; and “d” is the effect size (minimum clinically important difference).

### Data collection tools

#### Mindfulness-based intervention assessment questionnaire

This study used an electronic self-administered questionnaire containing the following four sections:

#### Part I: Basic participant characteristics

Researchers developed this part. It includes basic demographic and occupational characteristics such as age, gender, marital status, number of children, educational level, years of experience, and workplace.

#### Part II: Maslach burnout inventory

The Maslach Burnout Inventory-Human Services Survey for Medical Personnel MBI-HSS (MP) was developed by Maslach and Jackson [38]. We adopted MBI-HSS (MP) with permission from Mind Garden, Inc. (https://www.mindgarden.com/maslach-burnout-inventory/685-mbi-manual.html). The 22-item questionnaire assesses healthcare worker burnout syndrome frequency and severity. It evaluates three syndrome components. (a) Work-related emotional exhaustion (EE) is the feeling of being overwhelmed and emotionally drained (9 items). (b) Depersonalization (DP) is a lack of emotion and impersonal responses to the issue being addressed (5 items). (c) Personal accomplishment (PA) explains work efficacy and competence (8 items). 0 indicates “Never” and 6 indicates “Every day” on the 7-point Likert scale. Burnout is defined by high EE and DP levels, and low PA levels. Our MBI-HSS sample has a Cronbach alpha of 0.807.

#### Part III: Five-Facet Mindfulness Questionnaire (FFMQ)

The 39-item FFMQ [39] assesses participants’ general tendency to concentrate on daily activities based on five skills or factors. (a) Observation is the ability to observe and attend to internal and external experiences, whether sensations, emotions, or thoughts. (b) Description is labeling experiences with words. (c) Acting with awareness is the ability to consciously focus attention on each activity, rather than acting mechanically. (d) Non-judging of inner experience is non-evaluative or judging position-taking concerning the present experience. (e) Non-reactivity to inner experience is letting emotions flow without being pinned by them or ignoring them. FFMQ is a 5-point scale (from 1, indicating never or very rarely true, to 5, indicating very often or always true). Higher scores indicate more mindfulness, awareness, or attention. Our sample for FFMQ achieved a Cronbach alpha of 0.960.

#### Part IV: Self-compassion scale

This instrument, which was used to assess how an individual reacts to himself or herself in difficult situations, was developed by Kristin Neff [40]. It consists of 26 items with responses on a 1–5 scale. It measures six aspects of self-compassion. (a) Self-kindness is treating oneself with compassion and understanding when adversity or suffering occurs and acknowledging one’s love, happiness, and affection. This includes five items with a 25-point limit. (b) Self-judgment is acting hurtfully and critically toward oneself when faced with difficult circumstances. This included five items with a 25-point limit. (c) Common humanity is accepting mistakes as part of life. This included four items with a 20-point limit. (d) Isolation is the feeling of being alone or disconnected from others, especially in times of uncertainty, fear, imperfection, and weakness. This included four items with a 20-point limit. e) Mindfulness is when something unpleasant happens that requires clear and balanced awareness of the moment. This included four items with a 20-point limit. (f) Over-identification occurs as a depressive mood, obsessional tendencies, and a tendency to see everything as wrong. This included four items with a 20-point limit. Subscale scores were computed by averaging the responses to subscale items. Reverse-score of the negative subscale items, including self-judgment, isolation, and over-identification (i.e., 1 = 5, 2 = 4, 3 = 3, 4 = 2, 5 = 1), were obtained, and the mean was calculated to obtain the total self-compassion score. Our Self-Compassion Scale sample achieved a Cronbach alpha of 0.841.

### Intervention

The intervention group received eight 2.5-h MBI sessions over 2 months, whereas the control group received no intervention. The MBI was based on MBSR [22] and Self-Compassion Training for Healthcare Communities (SCHC) [41]. All sessions were live-streamed on Zoom in the evenings due to the COVID-19 pandemic limitations. The principles, benefits, and scientific evidence of mindfulness practice were discussed in an introductory session, followed by two brief practices. The participants indicated whether they wanted to join the 8-week training at the end of this session. Willing participants joined the WhatsApp group. Eight sessions with videos and interactive exercises were led by a mindfulness-trained researcher (second author) (Table 1).

**Table 1:** The description of the MBI sessions

Time	Theme	Content	Exercise	Home Practice
Week 1	**Introduction to mindfulness**	- Understanding the physiology of stress - Introduction to mindfulness and compassion-oriented practices (e.g. sitting, walking and breathing meditation) - Theoretical principles & scientific evidence - Possible advantages for CCNs	- Raisin exercise - Sitting meditation with focus on the breath	- Sitting meditation with focus on the breath - Routine activity - Eat one meal mindfully
Week 2	**Attention to breathing**	- Review home practices from previous session - Breath awareness meditation - Knowing attention anchoring - Knowing internal anchoring: breathing	- Practice mindful breathing - Encourage nurses to observe their breath and thoughts without judgement	- Mindful breathing
Week 3	**Attention to the body**	- Body perception - Effect of body posture upon sensations - Mindful awareness of breath, body sensations and movements: eating, walk - Enhance awareness to thoughts and feelings associated with food by practicing mindful eating	- Body scan meditation, where they scan their body from head to toe and release tension with each breath - Practice awareness to body sensation: pay attention to sensations in the body	Mindful eating: - Raisin exercise - Tasting fruits
Week 4	**Attention to thoughts**	- Review home practices from previous session - Curiosity, acceptance, and non-judgmental attitude - Introduce mindful hearing: listen to music - Discussion: what made us each have different responses to the same piece of music?	- Practice mindful breathing	- Practice mindful breathing
Week 5	**Self-care and compassion**	- Introduction to self-compassion - Compassion and loving kindness - Dispelling misgivings about self-compassion - Motivating self with compassion instead of criticism	- Loving-kindness meditation focused on self, where they prioritize providing positive energy to themselves and other people	- loving-kindness meditation - Body scan meditation - 3 min breathing space
Week 6	**Mindful Movement**	- Conscious movement, breathing observation and body tone - Conscious movement - Observation of body tone and breathing during movement	- 10 movement with full attention	- Mindful movement practice
Week 7	**Mindfulness in Daily Life**	- Introduction to mindfulness in daily life	- Mindful eating & walking exercise	- Eat one meal mindfully
Week 8	**Mindfulness Communication**	- Discussion: personal experiences in the past 6 weeks; ways to continue to cultivate mindful awareness in daily lives - Introduction to mindful communication - Reflection and conclusion of the MBSR program	- Mindful listening exercise	

Each session contains:

- A brief breathing exercise and relaxation, then a 30-min PowerPoint presentation on a related topic (e.g., dealing with suffering, and interpersonal relationships).
- A 45-min mindfulness and compassion workout. The intervention program included body scans, which involved focusing on different body parts (such as the toes, head, and back) and physical sensations (such as muscle tension or pain) at the moment, and breath awareness meditation, which involved focusing on the breath and observing how physical sensations change during inhalation and exhalation. Participants were taught about maintaining a mindset of acceptance, nonjudgmental, and equanimity, in addition to improving focus, cognition, memory, and control over one’s emotions. The mindful compassion sessions allowed participants to discuss their practice-related difficulties. The instructor then expressed her views.
- A 60-min group discussion on the subject matter and participants’ mindfulness practice experiences.

Five assignments were posted in the WhatsApp group for participants to practice in real life. Additionally, videos were available to guide meditation. Participants were given a written description of the activity and audio recordings for guided meditation and mindfulness exercises via WhatsApp every Saturday. Participants were sent a reminder to practice each day, which included a motivating word, image, or video related to the week’s topic. All adverse events reported by the trial participants were recorded. Participants who required additional psychological support were consulted by a psychologist at the Psychiatric Consultation Service of the Faculty of Nursing at Damanhour University, Egypt, either by their own initiative or at the trainer’s suggestion. Participants who required face-to-face counseling owing to their experience of temporary psychological issues triggered by their job demands received psychological counseling. Only two participants needed this type of counseling; they were monitored and followed up throughout the program’s implementation, and they expressed confidence in their capacity to resolve these urgent issues.

Nurses in the control group did not receive MBI training, and they were specifically informed that they were not supposed to participate in any mindfulness-related training during the study time. The control group received text message reminders via WhatsApp group to avoid mindfulness-related training during the study. They completed the questionnaires for the study both before and after the 8 weeks of MBI. Participants we reminded by the researchers to finish the questionnaire via text messages in the WhatsApp group. The content of the MBI training was distributed to nurses in the control group after completing the study.

### Data collection

All nurses in the study sites were contacted via WhatsApp and invited to take part in the study. The study included nurses who volunteered to participate. The study was conducted for 2 months (April–Jun 2021) in three phases after receiving approval from the appropriate institutional review board.

In Phase 1, researchers obtained the pre-intervention questionnaire data (Basic Participant Characteristics, Maslach Burnout Inventory, FFMQ, and Self-Compassion Scale) from eligible nurses in the control and intervention groups via a 1-h online survey form hosted on Microsoft Teams and sent to their email addresses or WhatsApp. These questionnaires were designed to evaluate the severity of burnout syndrome, mindfulness level, and self-compassion levels in response to caring for patients with COVID-19. To adopt the original translated Arabic version of the medical personnel MBI-HSS, permission was obtained from Mind Garden, Inc. (https://www.mindgarden.com/mind-garden-forms/61-translation-application.html). The Self-Compassion Scale and FFMQ questionnaires were translated into Arabic by the first and third authors, as well as interpreters. The back-translation technique was used for ensuring translation validity [42]. An experienced bilingual interpreter who was blind to the original English text was provided the Arabic translation for back into English translation. The first and third authors and five CCNs reviewed the English back-translation and made modifications where necessary. Five CCNs tested the updated Arabic version and were asked whether they had any problems completing the questionnaires. They did not report any concerns with comprehension of questionnaires; therefore, no revisions were required in the final translated tools.

Phase 2 consisted of eight MBI training sessions (one 2.5 h session per week). In Phase 3, post-intervention questionnaires (post-test) were administered to all nurses in the control and intervention groups to evaluate the effectiveness of MBI. The nurses were given instructions on completing the questionnaires independently.

### Validity and reliability

A panel of six CCN, psychiatric nursing, and mental health professionals used the Content Validity Index (CVI) to determine whether each instrument sufficiently covered the subject of interest. The CVI values were within acceptable limits. The study instrument’s average of CVI was 0.92 for Part I, 0.90 for Part II, 0.95 for Part III, and 0.91 for part IV. Additionally, the MBI’s training content was validated by two critical care and psychiatric nursing professors at the Faculty of Nursing, Damanhour University. Six nurses who provided direct care for patients with COVID-19 participated in a pilot study to evaluate the study tool’s clarity and reliability, which required no changes. The nurses who participated in the pilot study were excluded from this study.

### Statistical analysis

Data from this study were analyzed using the Statistical Package for the Social Sciences (IBM SPSS Statistics for Windows, Version 23.0, IBM Corporation, Armonk, New York). In this study, only those who completed the intervention and provided data at the follow-up were included in the analysis. The Kolmogorov–Smirnov test was used to verify the variable distribution normality, which revealed normally distributed data. Categorical variables are displayed as numbers and percentages. The chi-square test (Monte Carlo) was applied to compare categorical variables between groups. Normally distributed quantitative variables were compared between groups using the Student t-test, and pre- and post-intervention changes within each group were compared using the paired t-test. Cohen’s d was utilized for the determination of effect sizes, which was recommended with values of < 0.5, 0.5–0.8, and > 0.8 indicating effect sizes of small, medium, and large, respectively [43]. Differences with *p*-values of < 0.05 were regarded as statistically significant, while those with *p*-values of < 0.001 were regarded as highly statistically significant.

## Results

### Baseline sociodemographic and occupational characteristics of the study participants

**Fig. 1:**
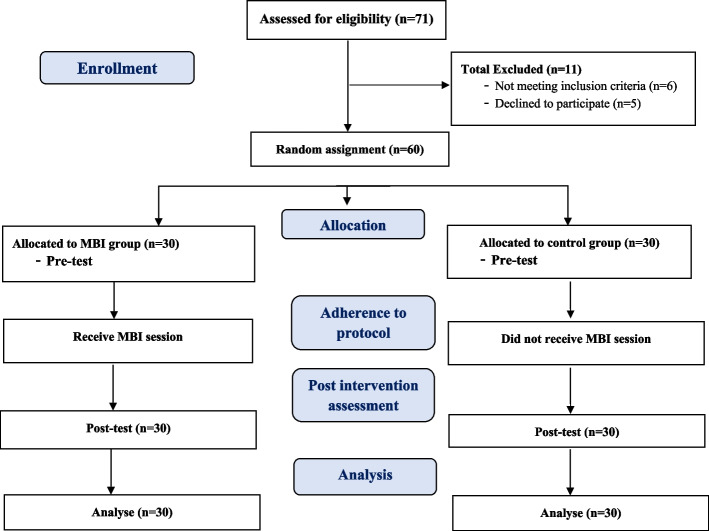
Study flow diagram of participants

The initial study sample included 71 participants. During the fieldwork, 11 participants were excluded, including 6 who could not continue engaging in the study and 5 who could not adhere to the training program. Thus, the final study sample of 60 participants, including 30 participants each in the MBI and control groups, was used in the analysis (Fig. 1). Table 2 describes participants’ sociodemographic and occupational characteristics in the MBI and control groups. Most of the participants were female, accounting for 70% and 80% of the MBI and control groups, respectively. Additionally, 56.7% and 63.3% of the MBI and control groups, respectively, were under 25 years old, with more than two-thirds of both groups having a bachelor’s degree. Most of the nurses in both groups worked in the respiratory ICU, accounting for 63.3% and 70.0% of the MBI and control groups, respectively. Participants’ working experience ranged from 1 to 3 years for 43.3% and 36.7% of the MBI and control groups, respectively. Study results reveal the comparability of the studied groups. No initial differences were found in baseline sociodemographic and occupational characteristics between the two groups.

**Table 2:** Sociodemographic and occupational characteristics in the MBI and control groups

Characteristic	MBI (*n* = 30)		Control (*n* = 30)		χ^2^	*p*
	**No**	**%**	**No**	**%**		
**Gender**						
Male	9	30.0	6	20.0	0.800	0.371
Female	21	70.0	24	80.0		
**Age**						
< 25 years	17	56.7	19	63.3	3.463	^MC^*p* = 0.378
25–29 years	8	26.7	7	23.3		
30–34 years	5	16.7	2	6.7		
> 35 years	0	0.0	2	6.7		
**Level of education**						
Nursing institute	7	23.3	8	26.7	0.759	^MC^*p* = 0.850
Bachelor degree	19	63.3	20	66.7		
Master degree	4	13.3	2	6.7		
**Years of experience**						
< 1 year	2	6.7	3	10.0	1.265	^MC^*p* = 0.773
1–3 years	13	43.3	11	36.7		
3-5 years	8	26.7	11	36.7		
> 5 years	7	23.3	5	16.7		
**Marital status**						
Single	25	83.3	22	73.3	0.884	0.347
Married	5	16.7	8	26.7		
**Place of work**						
Respiratory ICU	19	63.3	21	70.0	1.407	^MC^*p* = 0.7541
General ICU	11	36.7	9	30.0		
**Number of children**						
No	25	83.3	22	73.3	1.280	^MC^*p* = 0.657
1	3	10.0	6	20.0		
2	2	6.7	2	6.7		

*MBI* Mindfulness Intervention Group, χ^2^: Chi-square test, *MC* Monte Carlo

### Effect of MBI training on burnout, mindfulness, and self-compassion

**Fig. 2:**
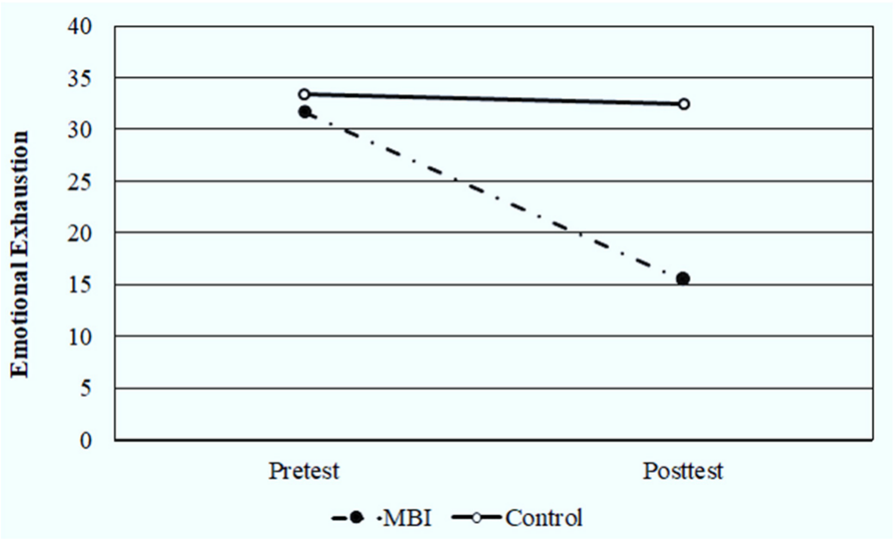
Comparison between MBI and control groups according to emotional exhaustion

The baseline pre-test mean scores for the EE, DP, and PA subscales did not initially differ significantly between the MBI group and control group, as shown in Table 3. The post-test mean score for EE revealed a significant (*P* < 0.001, Cohen’s d = 1.912) decrease for the MBI group (15.47 ± 4.44) as compared with control group (32.43 ± 8.87) (Fig. 2). Similarly, there was a significant (*P* < 0.001, Cohen’s d = 1.120) decrease in the post-test DP’s mean score (5.43 ± 3.68) for the MBI group compared with the post-test DP’s mean score (12.13 ± 5.98) for the control group. However, there was a significant (*P* < 0.001, Cohen’s d = 2.463) increase in the post-test mean score for PA (39.60 ± 3.70) in the MBI group as compared with the post-test mean score for PA (26.03 ± 5.51) in control group.

**Table 3:** Pre-test and post-test mean values of Burnout, Mindfulness, and Self-Compassion in MBI and control groups

Scale Subcategories	MBI Group							Control Group						Pre-test		Post-test		Cohen’s d (effect size)
	**Pre-test**		**Post-test**		**t**_**2**_	***p***	**Cohen’s d (effect size)**	**Pre-test**		**Post-test**		**t**_**2**_	***p***					
	**Mean**	**SD**	**Mean**	**SD**			**MBI group (Pre vs. Post test)**	**Mean**	**SD**	**Mean**	**SD**			**t**_**1**_	**p**	**t**_**1**_	***P***	**Post test (MBI vs. control)**
**MBI-HSS**																		
**Emotional Exhaustion**	31.77	8.88	15.47	4.44	11.041^*^	< 0.001^*^	4.101	33.37	10.68	32.43	8.87	0.418	0.679	0.631	0.531	9.366^*^	< 0.001^*^	1.912
**Depersonalization**	10.67	6.96	5.43	3.68	3.584^*^	0.001^*^	1.331	12.30	6.68	12.13	5.98	0.099	0.922	0.927	0.358	5.224^*^	< 0.001^*^	1.120
**Personal Achievement **^**a**^	25.87	8.33	39.60	3.70	10.370*	< 0.001*	3.851	25.30	5.21	26.03	5.51	0.491	0.627	0.316	0.753	11.202*	< 0.001*	2.463
**FFMQ**																		
**Observing**	25.93	3.81	27.93	4.0	2.484^*^	0.019^*^	5.268	24.53	2.99	24.17	3.16	0.446	0.659	1.582	0.119	4.046^*^	< 0.001^*^	1.190
**Describing**	23.93	2.29	30.0	2.80	8.671^*^	< 0.001^*^	0.923	24.67	4.16	23.27	3.31	1.544	0.133	0.847	0.401	8.501^*^	< 0.001^*^	2.033
**Acting with Awareness**	25.50	3.85	32.23	2.08	9.085^*^	< 0.001^*^	3.220	25.97	4.12	24.33	3.25	1.627	0.115	0.453	0.652	11.211^*^	< 0.001^*^	2.431
**Non-judging**	22.03	3.93	25.97	2.94	4.476^*^	< 0.001^*^	3.374	21.50	4.47	22.30	3.54	0.666	0.511	0.491	0.626	4.360^*^	< 0.001^*^	1.037
**Non-reactivity**	19.90	3.92	20.90	2.80	1.216	0.234	1.662	19.53	4.07	20.33	3.44	0.854	0.400	0.355	0.724	0.700	0.486	0.166
**Total FFMQ **^**b**^	**117.30**	**7.86**	**137.03**	**5.93**	14.184^*^	< 0.001^*^	**0.452**	**116.20**	**8.06**	**114.40**	**7.44**	**0.755**	**0.456**	**0.535**	**0.594**	13.027^*^	< 0.001^*^	**3.042**
**SCS**																		
**Self-kindness**	15.33	2.68	18.23	2.27	4.443^*^	< 0.001^*^	4.858	16.33	2.48	16.63	2.88	0.384	0.704	1.499	0.139	2.389^*^	0.020^*^	0.556
**Self-judgment**	14.0	3.14	17.57	1.61	6.410^*^	< 0.001^*^	1.650	15.27	3.27	16.23	2.56	1.319	0.197	1.531	0.131	2.417^*^	0.019^*^	0.523
**Common humanity**	12.50	1.96	15.20	1.69	6.723^*^	< 0.001^*^	2.381	11.70	2.20	12.03	2.53	0.619	0.541	1.487	0.142	5.780^*^	< 0.001^*^	1.253
**Isolation**	12.20	2.63	15.13	1.66	5.757^*^	< 0.001^*^	2.497	11.60	1.79	11.07	2.15	1.067	0.295	1.032	0.307	8.212^*^	< 0.001^*^	1.888
**Mindfulness**	13.20	2.96	14.30	1.37	1.699	0.100	2.138	12.87	2.29	12.83	2.12	0.070	0.944	0.488	0.214	3.185^*^	0.003^*^	0.693
**Over- identified**	10.83	2.21	14.07	2.05	5.747^*^	< 0.001^*^	0.631	11.60 ±	2.50	10.20 ±	2.23	2.269^*^	0.031^*^	1.258	0.214	6.984^*^	< 0.001^*^	1.735
**Total SCS **^**b**^	**78.07**	**4.57**	**94.50**	**3.83**	13.080^*^	< 0.001^*^	**2.134**	**79.37**	**4.57**	**79.0**	**4.57**	**0.295**	**0.770**	**1.102**	**0.275**	14.235^*^	< 0.001^*^	**3.392**

*MBI* Mindfulness Interventions Group, *MBI-HSS* Maslach Burnout Inventory-Human Services Survey for Medical Personnel, *FFMQ* Five Facet Mindfulness Questionnaire, *SCS* Self-Compassion Scale, ^a^ Scored in opposite direction emotional exhaustion and depersonalization, ^b^ Items with negative responses were reversed for total FFMQ & SCS score calculation, t_1_: Student t-test for comparing between study and control in each pre and post-intervention, t_2_: Paired t-test comparing between pre and post-intervention in each study and control, *: Statistically significant at *p* ≤ 0.05, Cohen’s d: Small effect at < 0.5, Medium effect at 0.5- < 0.8, Large effect at > 0.8

**Fig. 3:**
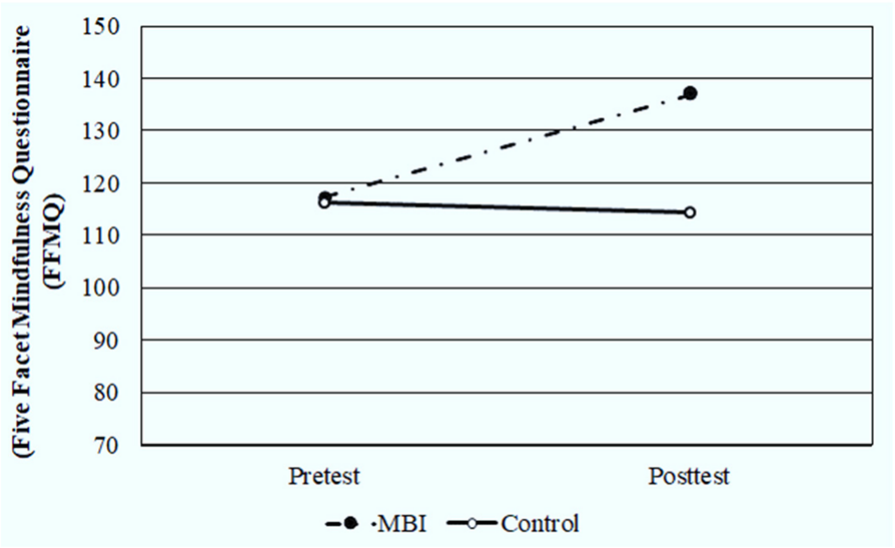
Comparison between MBI and control groups according to FFMQ

The FFMQ was used to measure mindfulness after the significant variations in MBI sessions. Table 3 illustrates that there was no significant difference (*p* = 0.594) in the pre-test total mean scores of the FFMQ for the MBI group (117.30 ± 7.86) as compared with control group. Comparing the post-test total mean score of the FFMQ for the MBI group (137.03 ± 5.93) with the control group (114.40 ± 7.44) represented a significant improvement (*p* < 0.001, Cohen’s d = 3.042) between them (Fig. 3). The separate analysis of the different subscales revealed that four of the five FFMQ facets (observing, describing, acting with awareness, and non-judging of inner experience) showed significant improvement (*p* < 0.001, Cohen’s d = 1.190, 2.033, 2.431, and 1.037 respectively), while the mean score for non-reactivity to the inner experience facet did not show significant differences between the studied groups following the MBI sessions (*P* = 0.486, Cohen’s d = 0.166).

**Fig. 4:**
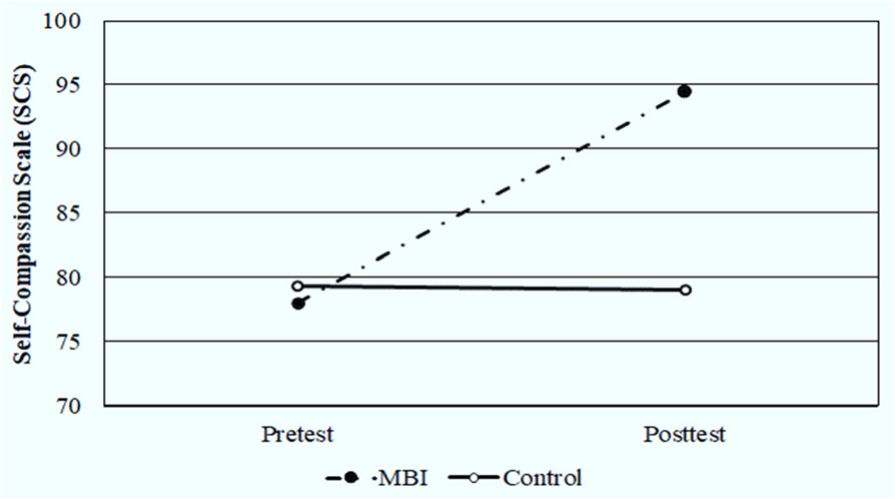
Comparison between MBI and control groups according to SCS

As shown in Table 3, there were no significant differences (*p* = 0.275) in the overall mean pre-test score of SCS between the MBI and control groups (78.07 ± 4.57 vs. 79.37 ± 4.57, respectively). While following the MBI training, there was a significant (*P* < 0.001, Cohen’s d = 3.392) increase in the overall mean post-test score of SCS for the MBI group (94.50 ± 3.83) as compared with control group (79.00 ± 4.57) (Fig. 4). Moreover, five of the six sub-categories of the SCS (self-kindness, self-judgment, common humanity, isolation, and over-identification) showed a significant (*p* < 0.001) improvement following the intervention for the MBI group. It can be noted from the same table that the MBI training has a positive effect on the total score of MBI-HSS subscales (EE, DP, and PA), FFMQ as well as SCS for the MBI group as evidenced by the large effect sizes detected by Cohen’s d.

## Discussion

According to recent studies, CCNs are likelier than other hospital workers to experience anxiety, depression, and burnout as a result of the COVID-19 pandemic [13, 16]. Numerous clinical studies have revealed that mindfulness and compassion interventions can reduce emotional distress and burnout among CCNs [36, 44, 45]. MBIs assist individuals to become aware of their thoughts, feelings, and body sensations in the present moment without judgment. This allows individuals to manage their emotions, minimize stress, and build resilience, which helps prevent burnout. Moreover, MBIs improve mindfulness and self-compassion while decreasing burnout. Mindfulness and self-compassion promote mental health by reducing the levels of anxiety, stress, and depression [46–48]. Considering the relationship between burnout, mindfulness, and self-compassion, it makes a theoretical reason for the present study to simultaneously examine the effects of MBIs on all three outcomes. When the effects of MBIs on various outcomes are investigated together, we can learn more about the potential advantages of these programs and the processes contributing to their efficacy. Additionally, recognizing how MBIs alter these outcomes may guide the development of interventions that are specifically designed for the requirements of CCNs. Therefore, this is one of the first studies that was conducted in Egypt to assess the effects of MBIs on burnout, mindfulness, and self-compassion among CCNs caring for patients with COVID-19.

Most mindfulness programs are 2.5-h group sessions focused on physical presence. Our MBI training sessions were online, allowing participants to practice at their own pace. Conducting classroom training was especially difficult for CCNs from work settings during the COVID-19 pandemic. Therefore, online training was beneficial because of its suitability for CCNs working in ICUs. In addition, our study established a WhatsApp group through which participants could communicate with one another and the instructor regarding their mindfulness practices. Most participants wanted to keep practicing mindfulness in their personal and professional lives. They enjoyed the format, length, and quality of the training materials.

Cohen’s d test displayed large pre-post differences in the mindfulness, self-compassion, and burnout scale scores in the MBI group. This study revealed that MBI improved three dimensions of burnout in nurses: EE, DP, and PA. The mean scores of EE,and DP significantly decreased after 8 MBI sessions, whereas the PA mean score significantly increased with large effect sizes. This beneficial effect of MBI could be attributed to the evidence that mindfulness helps individuals manage their well-being effectively by enhancing emotional understanding, acceptance, and the capacity to change or replace unfavorable mood states [49]. Additionally, these benefits may be due to physiological changes caused by MBI. MBI has reduced stress-induced physiological reactivity in healthcare providers (HCPs) by reducing salivary α amylase levels [50].

The current results of the study are consistent with Klatt et al. [51] and Ducar et al. [52] who revealed a significant decrease in burnout among HCPs after mindfulness training. Two Spanish randomized controlled trials by Aranda Auseron et al. [37] and Amutio et al. [53] indicated a significant reduction in the mean EE score as a result of MBI training for HCP. Gracia Gozalo et al. [44] investigated the effect of MBI on burnout, mindfulness, and self-compassion in ICU HCPs. They indicated that MBI training significantly reduced the EE mean score. Our results are consistent with those of Sarazine et al. [54], who demonstrated that MBI increases PA and decreases EE scores. However, Verweij et al. [55] revealed no significant difference in burnout between the study and control groups.

Moreover, this study showed that MBI sessions improved self-compassion in participants.The present study revealed that the mean SCS scores significantly increased after 8 weeks of MBI sessions in the MBI group compared with control group. A higher mean SCS’ subscales scores indicated that the MBIs group’s self-kindness, common humanity, and mindfulness had improved, while their self-judgment, isolation, and over-identification with negative ideas had decreased. This positive effect of MBI may be because mindfulness practices could enhance nonjudgmental self-awareness and emotional intelligence (awareness of oneself, others, and empathy) [56]. The awareness of one’s own needs, including physical, psychological, and emotional, is a component of mindfulness. Hence, the professional quality of life may be positively affected [57]. This awareness may have a positive effect on one’s response to workplace stressors. A recent review demonstrated that MBI reduces compassion fatigue and stress [58], thereby improving provider performance and well-being. The current study instructed participants from the beginning to direct their compassion toward themselves before extending it to others.

Perula-de Torres et al. [59] and Gracia Gozalo et al. [44] displayed similar results on the effects of a mindfulness program on physicians and nurses during the COVID-19 pandemic. They indicated the association between the mindfulness training program and the big change in the total mean Self-Compassion Scale scores of the intervention group compared with the control group. Neff et al. [41] and Franco and Christie [60] both demonstrated that the mindfulness training program made the intervention group more compassionate toward themselves than the control group. The Canadian study by Crowder and Sears [61] showed significantly increased Self-Compassion Scale scores of the intervention group and stayed high at weeks 13 and 26. Our self-compassion findings were contradicted by randomized controlled trial Verweij et al. [55], which indicated no significant difference in the mean Self-Compassion Scale scores between the MBI and control groups.

This study illustrated that the total mean score on the FFMQ significantly increased after 8 MBI training sessions in terms of the mindfulness level of the CCNs. Furthermore, this study detected significant improvement in the observing, describing, acting with awareness, and non-judging of inner experience aspects of the FFMQ following MBI sessions, whereas non-reactivity to inner experience did not significantly improved. These results are explained by the improvements in the Maslach Burnout Inventory’s three domains. Several studies showed that increasing CCNs’ PA levels improve observation, description, and non-reactivity to inner experience. CCNs with lower EE and DP are more aware and less judgmental. This nonjudgmental attitude promotes self-compassion. Additionally, MBI increases awareness and changes work and personal behaviors in most participants [62–65].

Amutio et al. [53] revealed highly significant differences in the global FFMQ scores and its aspects in the intervention group, except for the non-reactivity to the inner experience aspect. A Spanish-controlled trial conducted under the direction of Asuero et al. [66] revealed HCP who underwent mindfulness training experienced a significant improvement in three of the five FFMQ facets. However, inner experience description and non-judgment did not increase. Similarly, Suyi et al. [67] displayed that four parts of the FFMQ (observe, describe, do not judge, and do not react) significantly improved in the intervention group after MBI. Our results matched those of Aranda et al. [37], Perula-de Torres et al. [59], and Manotas et al. [68], who indicated that MBI sessions increased the total mean FFMQ score in the intervention group compared with the control group. In contrast, Gracia Gozalo et al. [39, 44] showed that their mindfulness program did not affect the FFMQ’s global mean score or its five facets in the intervention group compared with the control group. Additionally, Wang et al. [69] demonstrated that the mean FFMQ scores of the studied groups were not significantly different after program application.

### Limitations

There are certain limitations to this study that cannot be ignored. First, the small sample size and convenience sampling may limit the generalizability of the study results. Second, this study only included immediate post-intervention follow-up. Third, owing to COVID-19’s necessity of physical separation, online interventions are used instead of ones that are face-to-face. Online interventions lack face-to-face benefits, including rapport, nonverbal communication, and shared physical space. This may affect members’ engagement and belonging. Fourth, most of the participants were females, which may have influenced the sample’s representativeness and the generalizability of the findings. Female and male nurses are likely to react differently to MBIs. Fifth, the self-reporting utilized during the present study, increased the response bias for all study variables. Finally, our study used a completer analysis rather than an intent-to-treat analysis. Completer analyses may introduce selection bias in our study since the participants who completed the intervention and post-test assessments may differ from those who dropped out. This can further limit the generalizability of our findings. Due to practical and logistical issues, such as the inability to contact dropouts, we were unable to undertake an intent-to-treat analysis in this study.

## Conclusion

Overall, the findings of this study indicated that MBI was associated with a significant decrease in burnout and an increase in mindfulness and compassion among this sample of CCNs in Egypt. The findings suggest that MBI can be an efficient and practicable tool for enhancing the well-being of CCNs.

### Implications for clinical practice and future directions

The evidence gained from this study will add knowledge to critical care nursing practices about MBSR as an emotion regulation therapy that allows nurses to better face COVID- 19 pandemic. The incorporation of MBSR into critical care settings as an intervention for increasing self-care among nurses can decrease the negative effects associated with emotional exhaustion and compassion fatigue. Moreover, the practice of mindfulness will deliberate CCNs’ awareness of the present moment without passing judgment, which in turn lessens the compassion fatigue and nurses’ burnout and boosts patient and nurse satisfaction by providing effective and safe patient care. Further face-to-face randomized controlled trials with a larger sample size and long-term follow-up are recommended. To reduce the possibility of bias, future studies should consider using an intent-to-treat analysis. Moreover, It would be useful to compare self-reported questionnaire data with biological stress markers including salivary cortisol and oxidative stress/inflammatory biomarkers to support the psychological test.

## Data Availability

Due to confidentiality concerns, the data and materials used in the current study cannot be made publicly available. However, they are available from the corresponding author upon reasonable request.

## Abbreviations

ICU: Intensive care unit
COVID-19: Coronavirus disease 2019
MBI: Mindfulness-based intervention
CCNs: Critical care nurses
CCUs: Critical care units
PTSD: Post-traumatic stress disorder
HCWs: Healthcare workers
MBSR: Mindfulness-based stress reduction
FFMQ: Five-Facet Mindfulness Questionnaire
MBI-HSS (MP): Maslach Burnout Inventory-Human Services Survey for Medical Personnel
EE: Emotional exhaustion
DP: Depersonalization
PA: Personal accomplishment
HCPs: Healthcare providers
